# Internal noise sources limiting contrast sensitivity

**DOI:** 10.1038/s41598-018-20619-3

**Published:** 2018-02-07

**Authors:** Daphné Silvestre, Angelo Arleo, Rémy Allard

**Affiliations:** Sorbonne Université, INSERM, CNRS, Institut de la Vision, 17 rue Moreau, F-75012, Paris, France

## Abstract

Contrast sensitivity varies substantially as a function of spatial frequency and luminance intensity. The variation as a function of luminance intensity is well known and characterized by three laws that can be attributed to the impact of three internal noise sources: early spontaneous neural activity limiting contrast sensitivity at low luminance intensities (i.e. early noise responsible for the linear law), probabilistic photon absorption at intermediate luminance intensities (i.e. photon noise responsible for de Vries-Rose law) and late spontaneous neural activity at high luminance intensities (i.e. late noise responsible for Weber’s law). The aim of this study was to characterize how the impact of these three internal noise sources vary with spatial frequency and determine which one is limiting contrast sensitivity as a function of luminance intensity and spatial frequency. To estimate the impact of the different internal noise sources, the current study used an external noise paradigm to factorize contrast sensitivity into equivalent input noise and calculation efficiency over a wide range of luminance intensities and spatial frequencies. The impact of early and late noise was found to drop linearly with spatial frequency, whereas the impact of photon noise rose with spatial frequency due to ocular factors.

## Introduction

Contrast sensitivity varies substantially as a function of spatial frequency (SF) and luminance intensity^1–5^ and is limited by many internal factors including optical factors such as diffraction and aberrations of the optical system, stochastic absorption of photons by photoreceptors and neural factors such as stochastic neural activity. The current psychophysical study used an external noise paradigm to quantify the impact of various internal factors on contrast sensitivity as a function of SF and luminance intensity.

**Figure 1:**
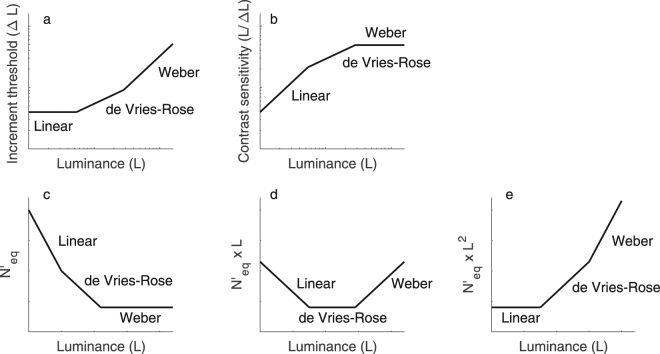
The three laws limiting contrast sensitivity and equivalent input noise. (**a**) The three laws represented with respect to the increment threshold in absolute units (Δ*L*). (**b**) The same three laws represented with respect to contrast sensitivity (*L*/Δ*L*). The second row represents the three laws limiting equivalent input noise in units where each law is independent of luminance intensity. (**c**) Weber’s law is independent of luminance intensity when equivalent input noise is plotted as a function of luminance intensity. (**d**) de Vries-Rose law is independent of luminance intensity when equivalent input noise multiplied by luminance intensity is plotted as a function of luminance intensity. (**e**) Linear law is independent of luminance intensity when equivalent input noise multiplied by squared luminance intensity is plotted as a function of luminance intensity. These graphs are represented on a log-log scale and (**c**) to (**e**) are in energy units.

**Figure 2:**
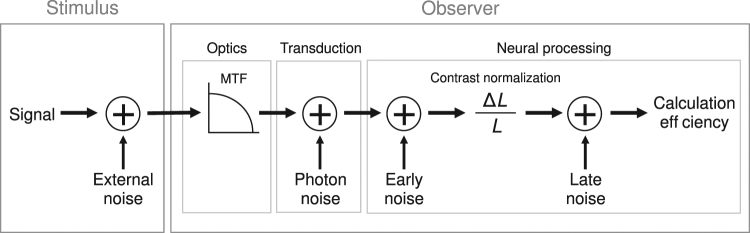
Observer model including the MTF, three additive internal noise sources and calculation efficiency. The additive internal noise sources comprise photon noise (i.e. phototransduction), early neural noise arising after optical aberrations modeled by the MTF and late neural noise arising after contrast normalization.

^4,18^), transduction process of photoreceptors modeled as photon noise added to the retinal image^14^ and the neural processing stage modeled as a combination of early noise occurring before contrast normalization, late noise occurring after the contrast normalization and calculation efficiency, which depends on the signal-to-noise ratio of the effective stimulus after the combination of all the noise sources^14^.

At low luminance intensities, increment threshold in absolute units (Δ*L*) is independent of the background luminance (*L*) (linear law in Fig. 1a), which corresponds to contrast sensitivity (*L*/Δ*L*) being proportional to the background luminance (Fig. 1b). This linear law can be explained by spontaneous neural activity occurring early in the visual system^6,7^ often referred to as the dark light of the eye^8^ (early noise in Fig. 2). At medium luminance intensities, increment threshold is proportional to the square root of the background luminance (de Vries-Rose law in Fig. 1a), which corresponds to contrast sensitivity being proportional to the square root of the background luminance (Fig. 1b). de Vries-Rose law can be explained by the stochastic fluctuations in the number of photon absorbed by the retina due to probabilistic absorption of photons^1,2^ (photon noise in Fig. 2). Indeed, photon absorption by the retina obeys the Poisson distribution and the number of photon absorbed varies according to Poisson’s law with a variance proportional to the luminance intensity and thus a standard deviation proportional to the square root of luminance intensity^9,10^. At high luminance intensities, increment threshold is proportional to the background luminance (Weber’s law in Fig. 1a), which corresponds to contrast sensitivity being independent of background luminance (Fig. 1b). Weber’s law can be explained by neural noise (e.g., spontaneous neural activity) located after contrast normalization^11–14^ (late noise in Fig. 2).

Thus, the variation of contrast sensitivity as a function of luminance intensity (three laws in Fig. 1b) can be explained by the fact that the impact of various internal noise sources varies differently with luminance intensity causing different internal noise sources to dominate at different luminance intensities: early spontaneous neural activity at low luminance intensities (linear law), probabilistic photon absorption at intermediate luminance intensities (de Vries-Rose law) and late spontaneous neural activity at high luminance intensities (Weber’s law). However, the impact of these internal noise sources also varies differently as a function of SF causing different internal noise sources to dominate and limit contrast sensitivity at different SFs. For instance, contrast sensitivity saturates (i.e., reach Weber’s law) at much lower luminance intensities for low SFs than for high SFs^4^. The impact of the internal noise can be psychophysically quantified relative to the impact of external noise (i.e., external noise paradigm^15^). Varying the contrast of external noise has a negligible impact on contrast threshold if the dominating noise source is internal and considerably affects contrast threshold when it is the dominating noise source. The impact of the internal noise can therefore be quantified relative to the amount of external noise having the same impact as the internal noise, namely, the equivalent input noise. To estimate the impact of the different internal noise sources, the current study used an external noise paradigm to factorize contrast sensitivity into equivalent input noise and calculation efficiency (LAM^14–16^). Given that calculation efficiency is independent of luminance intensity^16,17^, equivalent input noise (quantified in energy units) is inversely proportional to the squared contrast sensitivity and can therefore be, like contrast sensitivity, related to the three laws (linear, de Vries-Rose and Weber’s laws, Fig. 1c).

The aim of the current study was to characterize how the impact of the three internal noise sources (which varies with luminance intensity according to the three laws) varies with SF, and thereby identify the dominating one as a function of luminance intensity and SF (i.e. spatial-luminance domain). Equivalent input noise was therefore estimated as a function of a wide range of luminance intensities and SFs.

## Model

The observer model used in the current study is illustrated in Fig. 2 and is divided in three processing stages: optical factors represented by the modulation transfer function (MTF

The current study measured the equivalent input noise, which corresponds to the external noise level having the same impact as the total amount of internal noise in the system, that is, the sum of the three internal noise sources in our model. Given that the equivalent input noise is quantified in energy units (i.e., proportional to its variance), the impact of the combination of the three internal noise sources (i.e., equivalent input noise) is equal to the summation of the impact of the different internal sources. Being mainly interested in the neural factors (i.e. the three internal noise sources), let us consider the equivalent input noise at the entry of the retina (*N’*_*eq*_). Equation (1) represents the equivalent input noise in energy units at the entry of the retina quantifying the impact of the summation of the three sources of noise following the linear law, de Vries-Rose law and Weber’s law. *L* and *f* represent the luminance intensity and SF, respectively. The impact of photon noise (*N*_*photon*_) on equivalent input noise is inversely proportional to luminance intensity (i.e., de Vries-Rose law in Fig. 1c). Note that we mathematically defined *N*_*photon*_ as independent of luminance intensity and its impact on equivalent input noise (*N’*_*eq*_) is constrained to be inversely proportional to luminance intensity (*N*_*photon*_*/L*), which is mathematically equivalent to Pelli’s^14^ definition that photon noise is defined as inversely proportional to luminance. This noise being at the photoreceptor level is not spatially correlated and is therefore assumed to be spatially white^14,19^, and therefore constrained in our model to be independent of SF. The impact of early neural spontaneous activity (*N*_*early*_) on equivalent input noise (modeled as early noise in Fig. 2) is inversely proportional to squared luminance intensity (i.e. linear law, Fig. 1c). The impact of late neural spontaneous activity (*N*_*late*_) on equivalent input noise (i.e. late noise, Fig. 2) is independent of luminance intensity (i.e. Weber’s law, Fig. 1c). As a result, the equivalent input noise at the entry of the retina can be defined as a combination of these three internal noise sources:

1 $${{N}^{\text{'}}}_{eq}(L,f)=\frac{{N}_{photon}}{L}+\frac{{N}_{early}(f)}{{L}^{2}}+{N}_{late}(f).$$

However, the current study estimated the equivalent input noise at the entry of the eye (i.e. using a visual stimulus presented on a screen), not at the retina, so it was affected by the optical factors of the eye. The impact of most of the optical factors on contrast sensitivity can be characterized by the Modulation Transfer Function (MTF^4,18^), which represents the fraction of contrast transferred as a function of SF. Consequently, the contrast at the retina corresponds to the contrast at the input of the eye multiplied by the MTF. Thus, the external noise energy at the entry of the retina is equal to the noise energy at the entry of the eye multiplied by MTF squared. Because the equivalent input noise is quantified relative to the impact of the external noise (in energy units), the equivalent input noise at the entry of the retina (*N*_*eq*_′) is also equal to the equivalent input noise at the entry of the eye (*N*_*eq*_) multiplied by MTF squared:

2 $${N^{\prime} }_{eq}(L,f)=MT{F}^{2}(f)\times {N}_{eq}(L,f).$$

By combining equations (1) and (2), the equivalent input noise at the entry of the eye can be defined as a function of the MTF and the three internal noise sources:

3 $${N}_{eq}(L,f)=\frac{1}{MT{F}^{2}(f)}\times (\frac{{N}_{photon}}{L}+\frac{{N}_{early}(f)}{{L}^{2}}+{N}_{late}(f)).$$

As a result, this model of the equivalent input noise as a function of luminance intensity and SF depends on a scalar (*N*_*photon*_) and three functions with respect to SF (*MTF*(*f* ), *N*_*early*_(*f* ) and *N*_*late*_(*f* )). Note that no parameter in the model varies with respect to luminance intensity (*L*). To complete the model these three functions with respect to the SF need to be defined.

### MTF

Watson^20^ showed that the MTF can be well modeled with a generalized Lorentzian function:

4 $$MTF(f)={[1+{(\frac{f}{u(d)})}^{2}]}^{-a}\sqrt{D(f,d,555)},$$

with *u*(*d*) representing a second order polynomial depending on *d* the pupil diameter (here fixed at 3 mm), *D*(*f, d, 555*) representing the diffraction limited MTF depending on SF (*f* ), pupil diameter (*d*) and the wavelength of white light in focus at 555 nm (for details on *u*(*d*) and *D*(*f, d, 555*) see^20^). In his study, Watson found that an exponent *a* fixed to 0.62 fitted reasonably well the mean MTF of the 200 eyes tested in his study. In order to model the interindividual differences of the MTF, the current study used this generalized Lorentzian function (equation (4)) with the exponent *a* as a free parameter.

### Early noise

Early neural noise (*N*_*early*_(*f* )) represents spontaneous activity occurring before contrast normalization. To characterize how early noise varies with SF, four different functions were tested in the model: no early noise (early noise may not limit contrast sensitivity at any luminance intensities^21^), a constant (independent of SF), a linear function and a quadratic function, resulting in 0, 1, 2 and 3 degrees of freedom, respectively.

### Late noise

Late neural noise (*N*_*late*_(*f* ), i.e. spontaneous activity of neurons processing SF) is known to decrease with SF, which can be explained by the fact that the receptive field size decreases, and the cell density increases, with the cells preferred SF^19^. Specifically, if the receptive field width of simple cells is inversely proportional to its preferred SF and the cell density is proportional to the preferred SF, the impact of late noise would be expected to drop as a function of SF with a slope of −2^19^, which is consistent with previous observations^15,19^. To characterize how late neural noise varies with SF, three different functions were tested in the model: a linear function with a slope fixed to −2 (i.e. late neural noise inversely proportional to SF squarred^19^), a linear function with the slope as a free parameter and a quadratic function, resulting in 1, 2 and 3 degrees of freedom, respectively.

The global model fitting the equivalent input noise in the current study comprised one parameter for the MTF, one for photon noise, and the best functions for the early (0 to 3 parameters) and late neural noise (1 to 3 parameters).

## Results

### Contrast sensitivity

**Figure 3:**
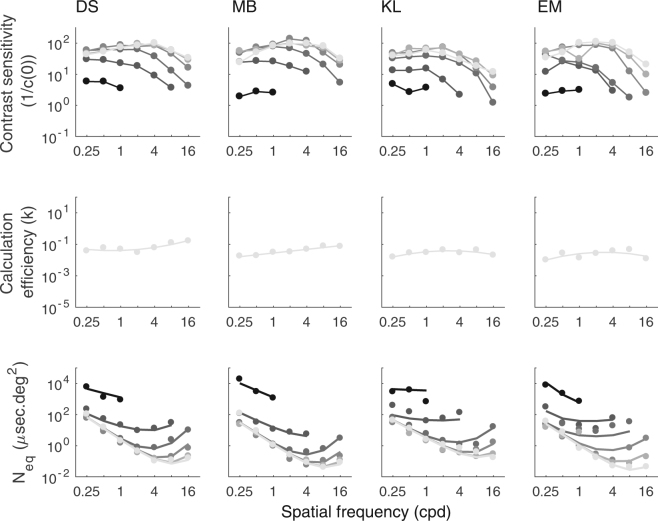
Contrast sensitivity function, calculation efficiency (k) and equivalent input noise (Neq). The first row represents contrast sensitivity of the four subjects (DS, MB, KL and EM) as a function of SF for different luminance intensities. The data points were connected with lines for clarity. The second row represents calculation efficiency estimated at 16261 Td for each subject (dots) and fitted with quadratic functions (lines). The third row represents equivalent input noise estimated for the whole range of luminance intensities (i.e. from 0.16 to 16261 Td) represented by dots and fitted with the best model (see Model section) represented by lines. Data are represented on a log-log scale and calculation efficiency and equivalent input noise are in energy units. Luminance intensities range in log steps from 0.16 to 16261 Td. The color gradation from the darkest to the lightest color represents the lowest to the highest luminance intensity, respectively.

**Figure 4:**
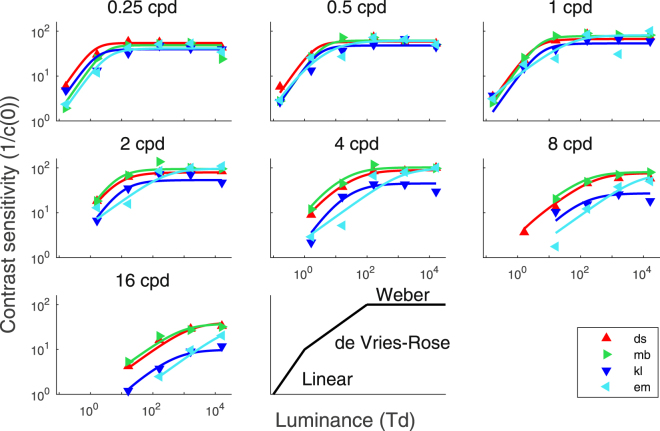
Contrast sensitivity as a function of luminance. Each graph represents the contrast sensitivity as a function of luminance of the four subjects for one SF on a log-log scale. Triangles (red, green, blue and cyan) represent the data for each subject and the lines represent the fit of the data with the best model (see Model section). The last graph (bottom right) represents reference lines for the linear law (slope of 1), deVries-Rose’s law (slope of 0.5) and Weber’s law (null slope). This representation of the three laws allows us to make an analogy with the data above.

Contrast sensitivity functions at different luminance intensities are shown in the first row of Fig. 3. As typically observed^4,21,22^, contrast sensitivity functions were band-pass at high luminance intensities and low-pass at low intensities. Contrast sensitivity gradually improved with luminance intensity until it reached saturation (Weber’s law). To illustrate the effect of luminance intensity on contrast sensitivity, the data are re-plotted as a function of luminance intensity in Fig. 4 separately for each SF. At low SFs (i.e. from 0.25 to 4 cpd), contrast sensitivity generally followed the linear law at low luminance intensities and Weber’s law at high luminance intensities. At high SFs (i.e. from 8 to 16 cpd), contrast sensitivity generally followed de Vries-Rose law at low luminance intensities and Weber’s law at high luminance intensities.

**Table 1:**
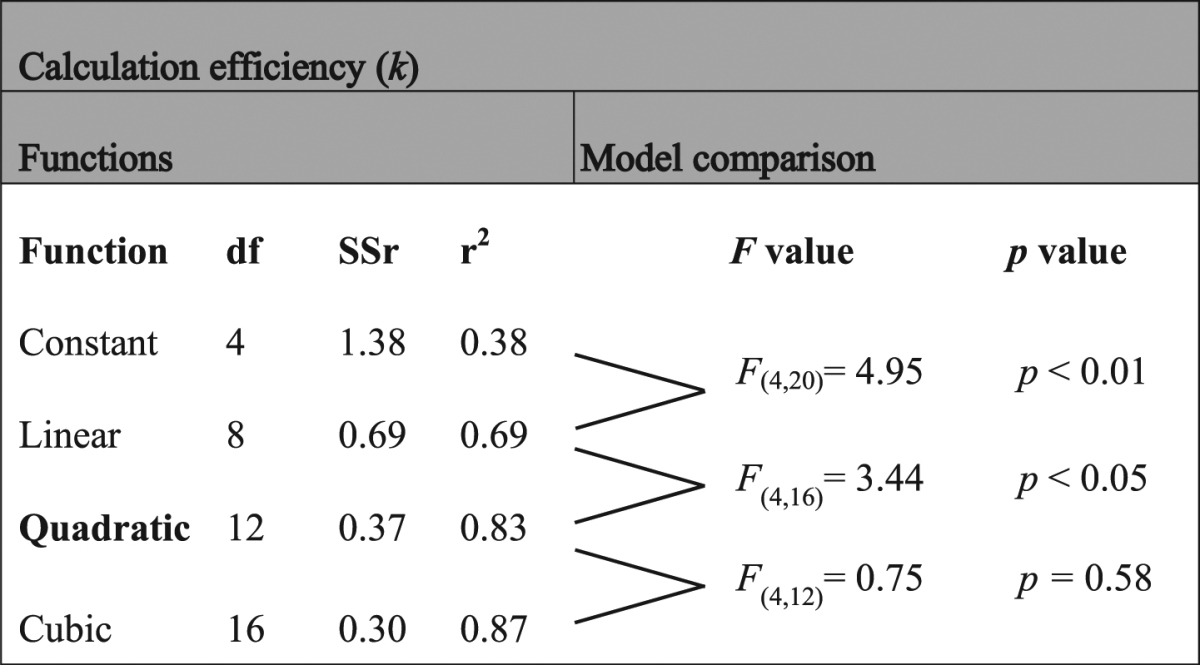
*F*-tests for the different calculation efficiency functions. Functions depending on SF tested to fit calculation efficiency are a constant, a linear function, a quadratic function and a cubic function. df is the degree of freedom of the function multiplied by the number of subjects (statistics at the group level). SSr is the sum of squared residuals and r^2^ is the coefficient of determination.

**Table 2:**
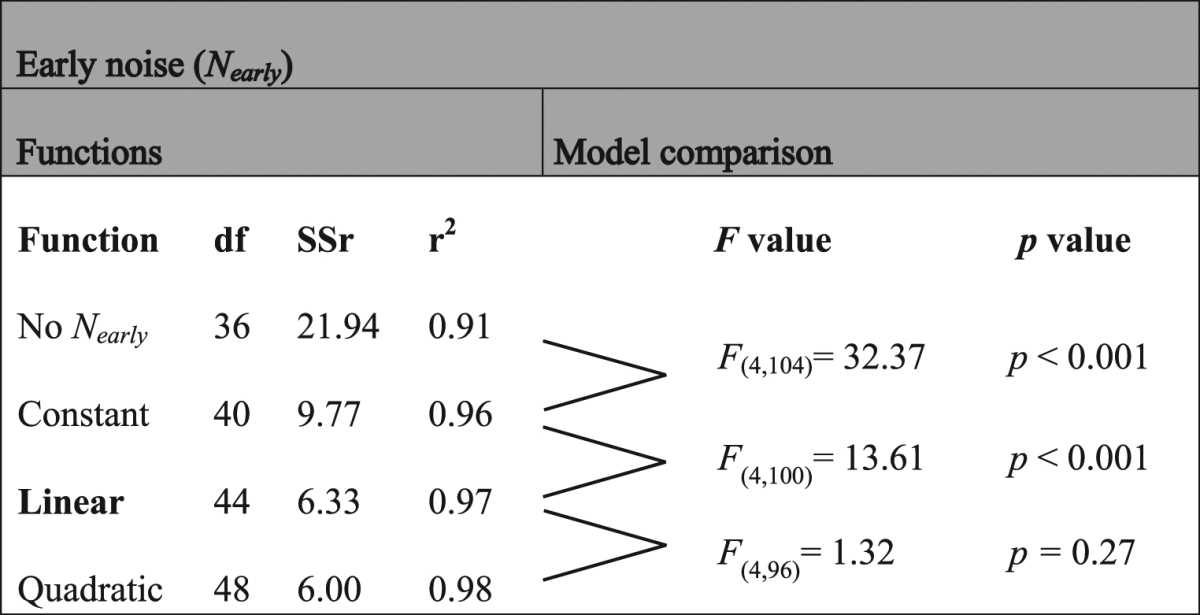
F-tests for the different early noise functions. Functions depending on SF tested to fit early noise are no early noise in the model, a constant, a linear function and a quadratic function. df is the degree of freedom of the fixed parameters in the model (i.e. 4 subjects × 9 parameters) plus the different functions defining the early noise (i.e. (0 to 3) × 4 subjects). SSr is the sum of squared residuals and r^2^ is the coefficient of determination.

**Table 3:**
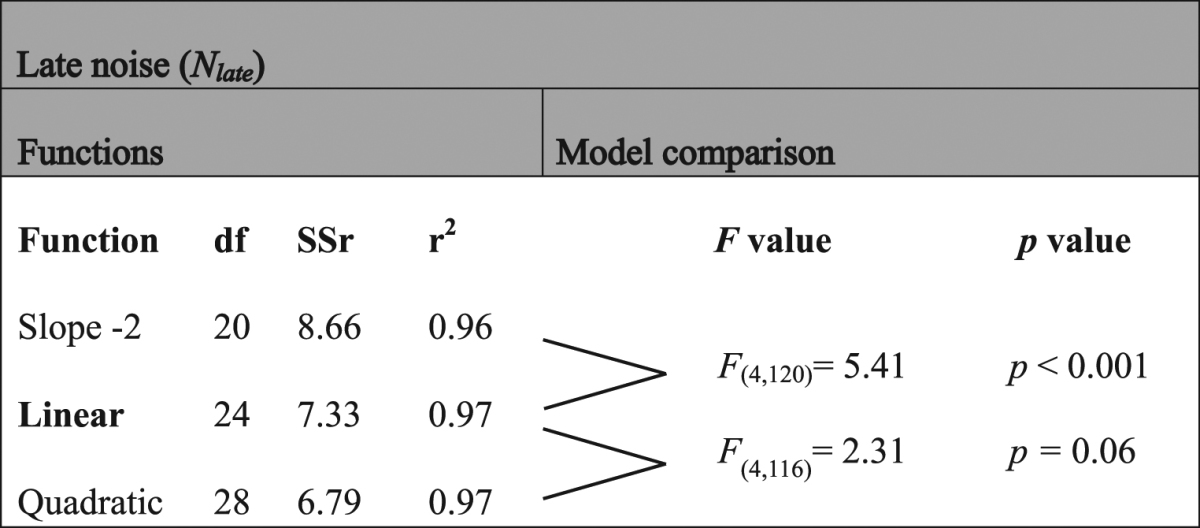
F-tests for the different late noise functions. Functions depending on SF tested to fit late noise are a linear function with a slope of −2, a linear function and a quadratic function. df is the degree of freedom of the fixed parameters in the model (i.e. 4 subjects × 4 parameters) plus the different functions defining the late noise (i.e. (1 to 3) × 4 subjects). SSr is the sum of squared residuals and r^2^ is the coefficient of determination.

### Calculation efficiency

Calculation efficiencies estimated at the highest luminance intensity based on energy thresholds in presence and absence of noise (equation (8)) are represented in the second row of Fig. 3. Model comparisons were performed to investigate the shape of the calculation efficiency function (Table 1). Analyses favored a linear function over a constant function (*F*_*(4*,20*)*_ = 4.95; *p* < 0.01) and a quadratic function over a linear function (*F*_*(4,16)*_ = 3.44; *p* < 0.05). This shows that the calculation efficiency was not independent of the SF.

### Equivalent input noise

Given the fitted calculation efficiency, equivalent input noise was estimated at various SFs and luminance intensities (bottom row of Fig. 3) using equation (9), that is, based on the measured energy thresholds in absence of noise and the fitted calculation efficiency, which was assumed to be independent of the luminance intensity. Statistical analyses regarding the shape of the early noise function rejected the absence of early noise and the constant early noise in favor of an early noise varying linearly with SF (Table 2). This shows that there were conditions under which early noise was the dominant noise source and this noise varied with SF. Statistical analyses regarding the shape of the late noise function rejected the linear model with a slope fixed to −2 in favor of a linear model with the slope as a free parameter (Table 3).

**Figure 5:**
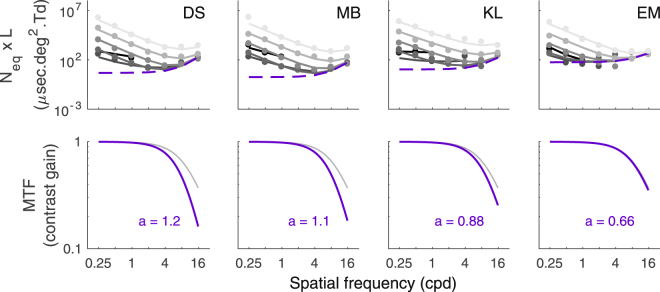
MTF estimation. The estimation of the equivalent input noise at the entry of the eye multiplied by luminance intensity (*N*_*eq*_** × ***L*) is represented in the first row of the graph. The impact of photon noise, which is independent of luminance intensity in these units, is represented by the lower limit (purple dashed line). Note that the impact of photon noise depends on the photon noise and MTF. The second row of the graph represents the MTF of each subject estimated by our model (purple line) and the MTF estimated by Watson’s model^20^ (grey line). The indicated values represent the value of the exponent of the general Lorentzian (equation (4)) found for each subject.

**Figure 6:**
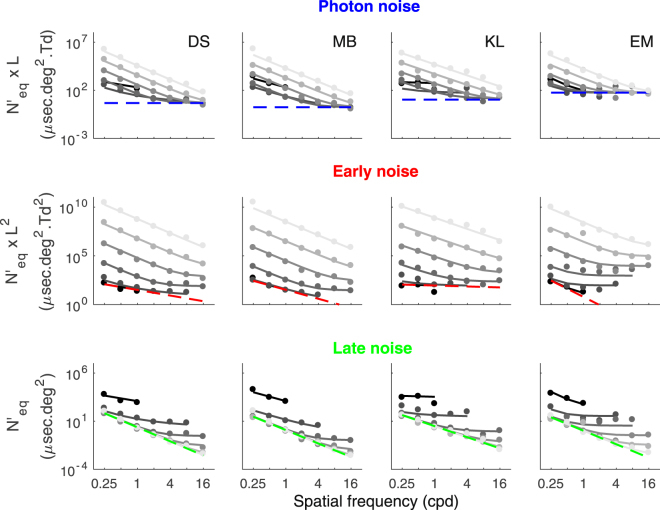
Equivalent input noise corrected for the MTF (N’_eq_). The equivalent input noise at the entry of the retina (*N’*_*eq*_) of each subject as a function of SF is represented in three different ways in order to represent the three sources of noise to be independent of luminance intensity. The first row represents *N’*_*eq*_ multiplied by luminance intensity and the lower bound (blue dashed line) is the photon noise estimated by our model. The second row represents *N’*_*eq*_ multiplied by luminance intensity squared and the lower bound (red dashed line) is the early noise estimated by our model. The third row represents *N’*_*eq*_ and the lower bound (green dashed line) is the late noise estimated by our model. The data for each luminance (in Td) is fitted with our model (grey gradation lines). The data is represented on a log-log scale and is in energy units.

In sum, the best model fitting the equivalent input noise as a function of luminance intensity and SF had 6 degrees of freedom: 1 parameter for the MTF, 1 parameter for the photon noise and linear functions for the early and late noise (2 parameters each). The fitting curves of the equivalent input noise in Figs 3, 5 and 6 correspond to the fit of this model.

To visualize the estimated MTF resulting from this global fit, Fig. 5 plots the equivalent input noise multiplied by the luminance intensity (

*N*_*eq*_x*L*). Based on equation (3), we get:

5 $${N}_{eq}(L,f)\times L=\frac{1}{MT{F}^{2}(f)}\times ({N}_{photon}+\frac{{N}_{early}(f)}{L}+L\times {N}_{late}(f)),$$

which shows that the impact of photon noise is independent of luminance intensity in these units (i.e. *N*_*eq*_x*L*; dash lines in top row of Fig. 5) and therefore sets a lower bound for the equivalent input noise: the impact of the sum of the noises at any luminance intensity cannot be less than the luminance-independent impact of only photon noise (see Fig. 1d). For low SFs at which photon noise was not the dominating noise source at any luminance intensities (only linear law and Weber’s law in Fig. 4), this lower bound was not reached at any luminance intensities. At high SFs, the fit rather suggests that photon noise was the dominating noise source over a wide range of luminance intensities (de Vries-Rose law in Fig. 4). Assuming that photon noise is spatially white^14,19^, the rise in equivalent input noise with SF would be due to the MTF of the eye. Note that although the MTF does not affect the photon noise *per se*, it affects the signal contrast and thereby affects the relative impact of photon noise (i.e. equivalent input noise). The bottom row of Fig. 5 is the classical representation of the MTF (i.e. contrast gain as a function of SF, equation (4)). The exponent of the general Lorentzian fitting the MTF slightly differed between the subjects and was similar to what would be expected from the literature (based on a large sample, a mean exponent of 0.62 was found^20^).

Given the MTF, equivalent input noise at the entry of the retina (*N’*_*eq*_, equation (2)) can be represented by factorizing out the impact of the MTF from the equivalent input noise at the entry of the eye (*N*_*eq*_). In other words, the impact of the three internal noise sources can be estimated after compensating for the effect of the MTF. To illustrate the impact of each noise source as a function of SF, equivalent input noises were plotted in units so that the impact of photon, early or late noise were independent of luminance intensity (*N′*_*eq*_*×L*, *N′*_*eq*_*×L*^*2*^ or *N′*_*eq*_, respectively, Fig. 1). Thus, in each of their respective units, the impact of a noise source was independent of luminance intensity and corresponded to a lower bound reached when it was the dominating noise source. Figure 6 represents the three noise sources to be independent of luminance intensity to highlight their relation with SF. As in the first row of Fig. 5, the first row of Fig. 6 represents photon noise as the limiting input noise, but now corrected for the optical aberrations (MTF). Therefore, photon noise was assumed to be spatially white (blue dashed line) as the rise in its impact was attributed to the MTF (Fig. 5). We can see that the data matching this lower bound (blue dashed line) are found at medium luminance intensities and high SF, thus for these conditions photon noise was the dominating internal noise source as expected from Fig. 4.

Early noise being inversely proportional to luminance intensity squared, its relation with SF is represented by the lower limit of the graphs on the second row of Fig. 6 (red dashed line). We can see that the data matching this lower bound was found at low luminance intensities and low SF as expected from Fig. 4. Thus, contrast sensitivity was limited by early noise in these conditions. Note that a substantial inter-subject variability was observed regarding the log-log slopes of the linear functions (i.e. −1.0, −1.5, −0.2 and −3.0 for subjects DS, MB, KL and EM, respectively), but the precision of these slopes are likely to be low due to the small number of conditions under which early noise was the dominating noise source (low SFs and low luminance intensities).

Late noise being independent of luminance intensity, its relation with SF is represented by the lower limit of the graphs in the last row of Fig. 6 (green dashed line). The inter-subject variability regarding the log-log slopes of the linear function modeling the late noise was relatively low (i.e. −2.4, −2.3, −1.9 and −2.3 for subjects DS, MB, KL and EM, respectively, mean of −2.2). We can see that the data matching this lower bound (green dashed line) was found at high luminance intensities and across the whole range of SFs, thus late noise was the dominating noise source in these

conditions.

## Discussion

Contrast sensitivity variations as a function of luminance intensity and SF were mainly due to variations in equivalent input noise as calculation efficiency is independent of luminance intensity and varied little with SF. Moreover, because calculation efficiency is independent of luminance intensity, the three laws characterizing contrast sensitivity as a function of luminance intensity (i.e. the linear law, de Vries-Rose law and Weber’s law) can be equally reformulated to characterize equivalent input noise as a function of luminance intensity (Fig. 1c; equation (1)) and therefore suggests the existence of three noise sources: photon noise, neural noise occurring before contrast normalization and neural noise occurring after contrast normalization. The advantage of relating these three laws directly to the equivalent input noise rather than contrast sensitivity was to determine how each of these noise sources varies as a function of the SF. Indeed, the contrast sensitivity variation with SF is caused by a variation in both calculation efficiency and equivalent input noise (Fig. 3). By factorizing out the calculation efficiency from the contrast sensitivity, the equivalent input noise can be independently investigated.

The model used in the current study to fit the data comprised early and late noise decreasing linearly with SF and photon noise being spatially white. Photon noise was the dominating noise at medium luminance intensities (1.6 to 162 Td) and high SFs (≥2 cpd), early noise was the dominating noise at low luminance intensities (0.16 to 1.6 Td) and low SFs (<2 cpd) and late noise was the dominating noise at high luminance intensities (>1626 Td) and across the whole range of SFs tested.

By measuring equivalent input noise across a wide range of SFs and luminance intensities, the current study could estimate the MTF, photon noise, early noise and late noise. While photon noise and late noise have been estimated in other studies^14,19^, early noise, to our knowledge, has never been estimated with an external noise paradigm. Nevertheless, contrast sensitivity has been studied as a function of luminance intensity and related to the three laws (de Vries-Rose, linear and Weber’s law). In most of these studies^4,23,24^ only de Vries-Rose law and Weber’s law were considered. Kelly^6^, on the other hand, considered all three laws, but observed the linear law only at high temporal frequencies, which were not evaluated in the current study. For static stimuli, Kelly found that contrast sensitivity followed Weber’s law at low SF and de Vries-Rose law at high SF at a luminance intensity of 50 Td, which is consistent with our results. In the current study, the spatial-luminance domain where early noise was the dominating internal noise source was low SFs (≤2 cpd) and low luminance intensities (≤1.6 Td).

The model that we develop in the current study is similar to the one developed by Rovamo, Mustonen and Näsänen^21^ which also takes into account the MTF of the eye, photon noise (or quantal noise; related to deVries-Rose’s law) and late noise (related to Weber’s law), but not early noise. An important difference between these models is that their model was applied to contrast sensitivity, whereas ours was applied to the equivalent input noise. As a result, they could not directly quantify how the impact of the internal noise sources varies with SF because contrast sensitivity depends on both equivalent input noise and calculation efficiency. Conversely, we were able to quantify how the impact of the internal noise sources varies with SF.

The current approach enabled us to estimate the MTF of the eye psychophysically. A similar approach has been used by Rovamo, Mostonen and Näsänen^25^ who also estimated the MTF by estimating equivalent input noise. However, the method they used relied on the assumption that photon noise was limiting contrast sensitivity at low luminance intensities, across all SFs. If photon noise was indeed limiting contrast sensitivity across the entire range of SFs tested, then the rise in equivalent input noise with SF would be due to the MTF (given that photon noise is spatially white^14,19^). However, the current study found that at low SFs and at low luminance intensities contrast sensitivity was limited by early noise, not photon noise, which implies that the impact of photon noise was less than the estimated equivalent input noise. Overestimating the impact of photon noise at low SFs reduces its estimated rise with SF, resulting in an underestimation of the drop of the MTF. This could explain their finding of a higher contrast gain for the MTF compared to other studies that estimated the MTF using other techniques.

At high luminance intensities (i.e.≥1626 Td), equivalent input noise was independent of luminance intensities across the whole range of SF tested (i.e. up to 16 cpd), which is consistent with Weber’s law and with noise arising after contrast normalization. To our knowledge, the current study estimated this late noise at high SF for the first time. A previous study^19^, for the same luminance range (i.e 1600 Td), did not reach Weber’s law at high SF and therefore could not estimate late noise for these SFs. This study rather found photon noise (i.e. noise at the photoreceptor level) at high SF suggesting that higher luminance intensities would have been required to reach saturation and thereby estimate late noise. We do not know what could explain these diverging results. Perhaps their subjects had greater photon noise relative to late noise (e.g. subject EM in the current study had relatively more photon noise than the others) or there is some difference in the experimental protocols. At this stage, any interpretation is speculative. What is clear in the current study is that contrast sensitivity reached saturation at the highest luminance intensities, even at high SFs (except for subject EM who did not reach saturation at high SFs), thereby enabling to estimate late noise across a wide range of SFs.

The late noise estimated in our study was found to drop with SF following a linear function with a slope of −2.2 (mean of the subjects), which is similar, but significantly different, from a slope of −2 found by Pelli^15,19^. Pelli suggested that a slope of −2 would be expected if neural density were inversely proportional to receptive field size and receptive field size were inversely proportional to squared SF. Our results are consistent with this explanation although one of these relations should be nuanced or other physiological mechanisms should be taken into account in order to reflect the slightly steeper slope found for the late noise.

In conclusion, the current study shows that the main variation in contrast sensitivity as a function of SF and luminance intensity can be explained by the fact that the impact of various internal noise sources varies with these parameters causing different internal noise sources to dominate in different conditions. More specifically, the equivalent input noise can be well modeled with a model having 6 degrees of freedom: 1 for the MTF of the eye, 1 for the photon noise and 2 for each of the two neural noise sources (early and late noise). Depending on the luminance intensity and the SF, contrast sensitivity variations could either be due to photon noise, early noise or late noise.

## Methods

### Observers

Four observers, aged from 23 to 34 years old (mean age = 27.75 years, SD = 4.57) with normal or corrected-to-normal vision participated in this study. The current study was approved by the Comité de Protection des Personnes Ile de France V, was carried out in accordance with the Code of Ethics of the World Medical Association (Declaration of Helsinki) and informed consent was obtained.

### Apparatus

All stimuli were generated by a homemade program and presented on a projector screen. The projector used was a LCD Panasonic PT-EW730Z with a refresh rate of 60 Hz and a resolution of 1280 × 720 pixels. Stimuli were presented at the center of a grey square of 37 × 37 cm having a mean luminance of 2300 cd/m^2^. Stimuli were viewed monocularly through a 3 mm artificial pupil at a distance of 48.5, 97, 194 and 388 cm from the screen depending on the SF tested. The projector was the only source of light in the room and was set behind the screen for a direct illumination. Luminance intensities of 2300, 230, 23, 2.3, 0.23 and 0.023 cd/m^2^ were obtained with neutral density filters with optical density of 0 (no filter), 1, 2, 3, 4 and 5, respectively. The output intensity of each color gun was carefully linearized using a Minolta spectroradiometer CS-1000. The Noisy-bit method^26^ implemented independently to each color gun, made the 8-bit display perceptually equivalent to an analog display having a continuous luminance resolution.

### Stimuli and procedure

An orientation discrimination task was carried out using a two-alternative forced-choice procedure (vertical or horizontal). Auditory feedback was given to the observer after each response by pressing one of two keys. Stimuli were sinusoidal gratings of one of seven SF (0.25, 0.5, 1, 2, 4, 8 and 16 cpd). The spatial window of the stimulus was a circular aperture with a diameter depending on its SF set to two visible cycles of the grating plus a half-cosine of half cycle. A black annulus centered on the stimulus and three times the size of the aperture (i.e. diameter equal to 6 periods of the grating) was continuously presented to minimize spatial uncertainty. Stimuli were presented for 500 ms.

The noise used was truncated-filtered noise^27^. Gaussian noise was spatially low-pass filtered with a cutoff two octaves above the signal frequency; its rms contrast was afterwards scaled to 25% and then truncated at 50%. The noise was resampled at 60 Hz. The resulting noise energies were 1200, 300, 76, 19, 4.7, 1.2 and 0.3 µs.deg^2^ for SF of 0.25 to 16 cpd, respectively. To avoid triggering a processing strategy shift, the noise was spatiotemporally extended (i.e. full-screen, continuously displayed^28–30^).

Contrast detection thresholds were measured using a 3down1up staircase procedure^31^ with a step size of a factor of 1.25 and were interrupted after 12 inversions. Such a staircase converged to a criterion level of 79% correct response corresponding to a *d*′ of 1.16. For each staircase, the threshold was estimated as the geometric mean of the last 10 inversions. Three staircases were performed for each condition. The threshold for each condition was estimated as the geometric mean of the three estimated thresholds.

For each SF and luminance intensity, two contrast thresholds were measured to estimate the equivalent input noise: one in absence of noise *c*(*0*) and the other in high noise *c*(*N*_*ext*_). To minimize luminance adaptation delays, the testing order was blocked with respect to the luminance intensity. Within each block of luminance intensity, the order of the SFs was randomized, but to minimized the displacement between testing distance, the three staircases for a given SF were performed one after the other. At low luminance intensities (i.e. 2.3 cd/m^2^ to 0.023 cd/m^2^), subjects adapted for 20 minutes in the dark (eyes closed) and contrast sensitivity at some high SFs was too low to be measured.

Given that calculation efficiency is independent of luminance intensity^16,17^ (also confirmed by a pilot study) contrast thresholds in noise were measured only at the highest luminance intensity (i.e. 2300 cd/m^2^). The energy threshold *E* (squared of the stimulus’ contrast function summed over space and time^15^ i.e. proportional to the squared contrast threshold) is known to be linearly related to the external noise energy *N*_*ext*_^15^. When the phase of the signal is unknown, this linear function can be represented as^32^:

6 $$E({N}_{ext})=\,\frac{{({d}^{\text{'}}+\sqrt{0.5})}^{2}}{k}({N}_{ext}+{N}_{eq}),$$

where *k* is the calculation efficiency. As a result, the energy threshold in absence of noise (*N*_*ext*_ = 0) is:

7 $$E(0)=\,\frac{{({d}^{\text{'}}+\sqrt{0.5})}^{2}}{k}{N}_{eq}.$$

Calculation efficiency (*k*) in energy units was calculated by combining equation (6) and (7):

8 $$k=\frac{{({d}^{\text{'}}+\sqrt{0.5})}^{2}{N}_{ext}}{E({N}_{ext})-E(0)}.$$

Given that calculation efficiency is independent of luminance intensity, the threshold in high noise (*E*(*N*_*ext*_)) and the calculation efficiency was estimated only at the highest luminance intensity (2300 cd/m^2^) for each SF. Based on equation (7), equivalent input noise was estimated for each condition based on the energy threshold in absence of noise (*E*(0)) and the calculation efficiency (*k*) estimated at 2300 cd/m^2^ for the given SF:

9 $${N}_{eq}=\frac{k}{{({d}^{\text{'}}+\sqrt{0.5})}^{2}}E(0).$$

### Analysis

In the present study, the model characterizing the equivalent input noise at the entry of the eye had four components: the MTF, the photon noise, the early noise and the late neural noise (see Model section for details). The different parameters relating to each of these components were independently defined for each subject. The MTF and photon noise were each estimated with only one parameter. For early and late neural noise, different functions were tested. The statistical analysis to determined the best fitting model for early and late neural noise was performed at the group level in order to define the general trend of the shape of these functions. In other words, the best fitting model was constrained to be the same for all subjects (i.e. same number of free parameters), but the value of each free parameter was independent across subjects.

The estimation of early and late noise being relatively independent one of the other (i.e. each dominating in a different luminance range), finding the best fitting function for each noise can be analyzed separately. The best fitting function was first analyzed for the early noise. For this analysis, the late noise was fitted with seven independent variables (one parameter for each SF). Given that the early noise was modeled with zero to three free parameters per subject (i.e. no early noise, a constant, a linear function and a quadratic function respectively), the different tested models comprised nine to twelve free parameters per subject (i.e. one for the MTF, one for the photon noise, seven for the late noise and zero to three for early noise). Model comparisons using *F*-tests enabled to determine the number of free parameters that were statistically justified for the early noise.

Afterwards, to analyze the best fitting model for the late noise, the early noise was modeled with the best fitting model found previously (linear function, i.e. 2 free parameters, see result section). Given that the late noise was modeled with one to three free parameters per subject (i.e. a linear function with a slope fixed to −2, as suggested by Raghavan^19^, a linear function and a quadratic function, respectively, see Model section), the different tested models comprised five to seven free parameters per subject (i.e. one for the MTF, one for the photon noise, two for the early noise and one to three for late noise). Model comparison with *F*-tests enabled to determine the number of free parameters that were statistically justified for the late noise.

To determine the number of free parameters statistically justified to fit the calculation efficiency as a function of the SF, *F*-tests were also performed. The different models tested were a constant (independent of SF), a linear function, a quadratic function and a cubic function.

All analyses were performed in log units. This fitted calculation efficiency using the best fitting model was used for the estimation of the equivalent input noise (equation (9)).

### Data availability

The data that support the findings of this study are available from the corresponding author upon reasonable request.
